# Evaluating Vascular Depth-Dependent Changes in Multi-Wavelength PPG Signals Due to Contact Force

**DOI:** 10.3390/s24092692

**Published:** 2024-04-24

**Authors:** Joan Lambert Cause, Ángel Solé Morillo, Bruno da Silva, Juan C. García-Naranjo, Johan Stiens

**Affiliations:** 1 Department of Electronics and Informatics (ETRO), Vrije Universiteit Brussel (VUB), 1050 Brussels, Belgium; angelsm@etrovub.be (Á.S.M.); bruno.da.silva@vub.be (B.d.S.); jstiens@etrovub.be (J.S.); 2 Department of Biomedical Engineering, Universidad de Oriente, Santiago de Cuba 90500, Cuba; 3 Centre of Medical Biophysics, Universidad de Oriente, Santiago de Cuba 90500, Cuba; jcgnaranjo@uo.edu.cu

**Keywords:** photoplethysmography, multi-wavelength PPG, contact force, signal morphology, skin viscoelasticity

## Abstract

Photoplethysmography (PPG) is a non-invasive method used for cardiovascular monitoring, with multi-wavelength PPG (MW-PPG) enhancing its efficacy by using multiple wavelengths for improved assessment. This study explores how contact force (CF) variations impact MW-PPG signals. Data from 11 healthy subjects are analyzed to investigate the still understudied specific effects of CF on PPG signals. The obtained dataset includes simultaneous recording of five PPG wavelengths (470, 525, 590, 631, and 940 nm), CF, skin temperature, and the tonometric measurement derived from CF. The evolution of raw signals and the PPG DC and AC components are analyzed in relation to the increasing and decreasing faces of the CF. Findings reveal individual variability in signal responses related to skin and vasculature properties and demonstrate hysteresis and wavelength-dependent responses to CF changes. Notably, all wavelengths except 631 nm showed that the DC component of PPG signals correlates with CF trends, suggesting the potential use of this component as an indirect CF indicator. However, further validation is needed for practical application. The study underscores the importance of biomechanical properties at the measurement site and inter-individual variability and proposes the arterial pressure wave as a key factor in PPG signal formation.

## 1. Introduction

Photoplethysmography (PPG) is a non-invasive, cost-effective optical technique that measures blood volume changes. The signal comprises a small AC component superimposed on a slowly varying signal (DC) [1]. While the DC component reflects tissue absorption and variations from respiration, thermoregulation, and sympathetic activity, the AC component represents blood volume changes in each cardiac cycle. It has been successfully proven in various applications, such as measuring heart and respiratory rates, evaluating arterial and venous diseases, and estimating arterial oxygen saturation (SpO_2_) [2].

The PPG signal is a rich source of information that can be used to infer various physiological parameters [3,4]. For instance, the AC/DC ratio represents the pulsatile amplitude to basal signal level, which sheds light on pulsatile variability and steady tissue perfusion [5]. Analyzing the area under a PPG signal cycle can help estimate pulsatile blood volume [6]. On the other hand, cycle length, or the interval between two systolic peaks, inversely correlates with heart rate (HR) [7]. Likewise, the width of the systolic peak can provide insights into pulse wave velocity and wave reflections [8]. Similarly, the relationship between the systolic and diastolic wave amplitudes can indicate arterial stiffness (AS), and the signal transition time from the diastolic to systolic peaks reveals circulatory dynamics [9]. Additionally, the first and second derivatives of the PPG can pinpoint inflection points in the cardiac cycle, offering further detailed insights [10].

Deriving physiological parameters from a PPG signal requires good-quality signals and proper analysis, especially with the growing interest in wearable devices and telehealth applications. Precise PPG data interpretation is usually affected by factors that influence the PPG signal, including the properties of the emitted light [11], interference from ambient light [12], the sensitivity of the photodetector [13], the choice of the measurement point [14], the ambient temperature [15], and, notably, contact force (CF) [16], a factor that is frequently underestimated [17,18].

Multiple studies indicate the need to standardize PPG sensor design to guarantee reliable data analysis [16,19,20]. Variability in CF can alter PPG signals, leading to potential misinterpretations of physiological parameters. As the adoption of PPG-based devices expands, especially in the wearable domain, it is critical to understand and counteract the effects of CF to ensure reliable data collection, optimized sensor design, and enhanced clinical decision-making. As pointed out by May et al. [21], there remains uncertainty about how CF affects PPG and which specific signal features are most susceptible to these changes, making it an ongoing area of exploration. This uncertainty is accentuated in multi-wavelength photoplethysmography (MW-PPG), where the effects of CF at specific wavelengths have not yet been exhaustively investigated.

In this research, the main objective is to elucidate specific response patterns to CF variations on MW-PPG signals. Data collection involves recording five wavelengths (470, 525, 590, 631, and 940 nm), CF, and temperature from the right and left index fingertips of 11 volunteers. This process is carried out under controlled conditions encompassing both increasing and decreasing phases of CF. The investigation begins with analyzing unprocessed MW-PPG signals, followed by a comparative study across various wavelengths to discern distinct response patterns attributable to CF variations. The impact of CF on the DC trend of MW-PPG signals is examined, and the underlying correlation is explored. Additionally, alterations in the AC component are investigated, and the modulation of AC pulse contours during both increasing and decreasing phases of CF is studied. This comprehensive approach intends to deepen our understanding of MW-PPG signal behavior under different CF levels to advance toward more accurate and reliable cardiovascular monitoring techniques.

The primary contributions of this work are as follows:

- We provide a novel multi-parametric dataset integrating synchronized MW-PPG, CF, and skin temperature measurements for comprehensive data analysis;
- We explore the integral relationship between CF variations, tissue mechanics, and MW-PPG signal evolution;
- We give a preliminary examination of the correlation between the CF and the DC component of the MW-PG signal.

The paper is structured as follows: It begins with exploring related work in Section 2, which provides a review of previous research. Then, Section 3 details the characteristics of the methodology and the process of collecting and analyzing experimental data. The research results are presented in Section 4. Following this, Section 5 delves into the analysis and interpretation of these results, exploring their meaning and implications in the field of study. Section 6 outlines potential avenues for subsequent studies. The paper concludes with a summary in Section 7.

## 2. Related Work

In addition to the pioneering research conducted by Dassel et al. [22] in the 1990s, who studied the effects of CF on reflectance pulse oximetry, particularly in the forehead, with the hypothesis that applying pressure could enhance measurement precision, there have been other significant studies in this area. Dassel et al. hypothesized that applying pressure could improve the accuracy of these measurements, and their research results substantiated this belief. Notably, the work of Teng and Zhang [19] further investigated the impact of CF on fundamental changes in an 880 nm PPG signal. Their findings indicated that specific PPG parameters, such as the DC amplitude, increased as the contacting force increased, while the normalized pulse area decreased. Simultaneously, the pulse amplitude and the ratio of AC/DC initially demonstrated an increase but subsequently declined. It was observed that most subjects reached peak pulse amplitude with a force range of 0.2–0.4 N. Furthermore, this study underscored the importance of considering variations in finger temperature among subjects, as these variations could also influence PPG readings.

Further contributing to this field, Rafolt and Gallasch [23] focused on how different pressure levels in a PD affected the detection of the arterial pulse using a 940 nm wavelength. Their experiment utilized an elastic ring as a spacer between the skin and the sensor, creating higher localized pressure. This setup resulted in an enhanced AC component under low-force conditions. This study emphasizes the relationship between the sensitivity of the pulse signal to motion artifacts and the slope of the DC component. It demonstrates how using an elastic ring can enhance this sensitivity. Additionally, several studies have focused on how the CF between the PPG sensor and the measurement site influences pulse transit time (PTT) measurements. The PTT is essential for evaluating arterial viscoelastic properties associated with AS and may indicate early cardiovascular disease. Within this scope, the works of Teng et al. [24,25] and Chandrasekhar et al. [26] are particularly notable. These stress the relevance of considering the mechanical properties of the tissue as well as the viscoelasticity and non-linear elasticity of the finger artery, providing a more holistic and precise approach to interpreting PPG measurements.

A follow-up study by Grabovskis et al. [27] pointed out that inappropriate sensor CF can adversely affect the AC PPG second-derivative peak ratio (b/a), emphasizing the importance of CF in the interpretation of PPG signals and the estimation of AS. The b/a parameter varied greatly with changes in CF (>300%) but was highly repeatable (variability coefficient < 5%) at the optimal CF. These results correlated with the arterial pressure–volume model, suggesting that certain characteristics of the PPG signal are optimized at specific contact pressures. In the experiment, the optimal CF was consistent and repeatable, underscoring the importance of controlling CF when interpreting clinically relevant features of the PPG. Alternatively, Spigulis et al. [28] investigated PPG signals relative to different wavelengths (405, 532, 645, 806, and 1064 nm). The results show that occlusion pressures were lower for shorter wavelengths, which are associated with superficial layers of the skin. Unique patterns were identified at specific wavelengths, such as 405 nm, which showed occlusions at a load of 430 g. As the probe charge varied, signals with different wavelengths reacted differently. Despite having the deepest penetration, the 1064 nm signals often aligned with features of the shallowest wavelength, 405 nm, showing the inherent complexity of MW-PPG signals.

Continuing this exploration, May et al. [21] analyzed the impact of the CF on the PPG signal morphology. Using an in vitro approach with a vascular tissue phantom miming human anatomy, the research identified an optimal sensor contact pressure range between 35.1 mmHg and 48.1 mmHg. Only red (660 nm) and infrared (940 nm) wavelengths were used. The study discerned that while time-based PPG features like cycle duration remain relatively unaffected by sensor CF, amplitude-related attributes, such as pulse amplitude and cycle area, are more susceptible. These amplitude features, instrumental in applications from pulse oximetry to blood pressure (BP) assessments, are derived from intricate interactions of stroke volume, vascular compliance, and tissue congestion. Hence, any deviation in CF can skew evaluations, leading to potential inaccuracies.

Additionally, the study noted that red PPG signals displayed more consistency across experiments, suggesting either noise resilience due to dye absorption or the inherent stability of red signal features. From a physiological perspective, the study delved into arterial behavior under external forces exerted by PPG sensors, highlighting the dual function of arteries: conduit and cushioning. The arterial walls, always pre-stressed due to BP, exhibit a unique response when external forces, like those from PPG sensors, are applied. A balance between external and internal pressures can maximize PPG signal amplitude as the arterial wall stress minimizes. This intricate balance and its implications on PPG readings reinforce the importance of optimal sensor CF to ensure accurate readings. Despite the controlled environment and the effort to replicate human physiological conditions, it is important to note that these findings are based on an in vitro model.

The analysis of related studies clearly illustrates the complex response of the PPG signal to CF. Such studies also underscore the need to investigate this phenomenon further. Variations in results, measurement points, and methods to quantify force-related values highlight individual variability as a crucial factor. These investigations predominantly aim to identify an optimal CF value and reveal significant wavelength-dependent differences. However, the lack of consideration of biomechanical skin properties usually related to age, gender, body mass index, and individual physiological characteristics suggests a gap in existing research. This oversight raises further questions about the influence of CF on PPG signals, especially within the context of MW-PPG, indicating the need for a more exhaustive approach.

The following sections will comprehensively explore how variations in CF impact the morphology of MW-PPG signals at the fingertips, contributing to bridging the identified gap in current research and paving the way for a deeper understanding of this interaction.

## 3. Materials and Methods

### 3.1. Experimental Platform

**Figure 1:**
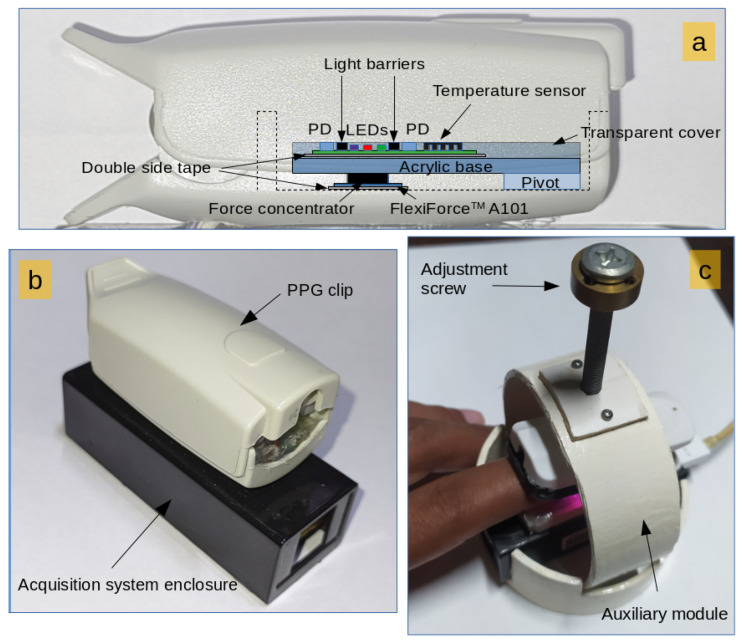
Details of the experimental platform. (**a**) Sensor layout. (**b**) Acquisition module. (**c**) Auxiliary module.

The experimental platform comprises two primary modules: the acquisition module and the auxiliary module. The acquisition module is based on previous work [29]. Figure 1 illustrates the experimental setup of the measurement system. Optical, temperature, and CF sensors are placed within the housing of an oximetry clip. Figure 1a shows the internal details of the sensor layout. The PCB supporting the sensors is fixed to the clip by employing a pivot, allowing free force transmission to the CF sensor (Tekscan Inc., Norwood, MA, USA, FlexiForce A101). The CF sensor is located on the bottom inner surface of the finger clip. A cylindrical force concentrator with a slightly concave finished surface covered by a thin transparent layer ensures even force distribution and easy cleaning.

The main board of the acquisition module is located in a plastic case. The plastic case is adhesively bonded to the finger clip for enhanced durability and seamless electrical connection between the PCBs (Figure 1b). The auxiliary module is crafted from polyvinyl chloride (PVC) and offers free control over the exerted force on the fingers. An adjustment screw delivers pressure to the top of the acquisition module, and a spring at the rear of the clip ensures the clamps remain open when not subjected to external pressure. Figure 1c shows the configuration adopted for the measurements, where the acquisition module is inserted into the auxiliary module.

Five distinct wavelengths (470, 525, 590, 631, and 940 nm) were employed for MW-PPG measurements. The distances between the PD and the LEDs were 2.5 mm for 470 nm and 525 nm, 3.8 mm for 590 nm, and 4.5 mm for 631 nm and 940 nm. The peak wavelength of the LEDs was obtained by setting the bias current to 20.0 mA. All signals were captured using an ADC resolution of 16 bits and a sampling rate of 100 Hz except for temperature, which was recorded at a sampling rate of 1 Hz. A USB connection to a laptop powered the system, providing real-time signal feedback and serving as a data transmission medium. During the measurements, the computer was not connected to the electrical network to guarantee primary electrical isolation.

### 3.2. Data Collection

The study was conducted in compliance with the Declaration of Helsinki and was approved by the Ethics Committee of the Dr. Juan Bruno Zayas General Teaching Hospital in Santiago de Cuba, Cuba. Eleven healthy participants (8 males and 3 females) with an average age of 32.1 ± 8.2 years provided informed consent. Key demographic data, including gender, age, and skin tone, were documented, with skin tone assessed using the Fitzpatrick scale.

Measurements were conducted in a climate-controlled room at 27 ± 2 °C and 75 ± 5% relative humidity, with data acquired from the index fingertips, starting with the left hand and then the right. Due to technical limitations, data from the left hand of subject No. 11 were not obtainable, resulting in a total of 21 data records (10 from the left hand and 11 from the right). To ensure the confidentiality of volunteer identities, each subject was assigned a unique ID for indexing and referencing. Experimental data were encoded using this ID, with a letter indicating the hand (‘R’ for right and ‘L’ for left) and the test type. For example, data from the right and left index fingers of subject No. 8 are coded as SR8 and SL8, respectively. The average duration of measurements was 21.84 ± 6.09 min for the left hand and 21.10 ± 6.36 min for the right, adding up to approximately 7.5 h of multi-parametric MW-PPG recordings. A summary of the basic information of the subjects is available in Table 1.

Instructions were given to lie comfortably in a supine position on a bed. A relaxation period of 10 min was allocated to allow for breathing adjustment and environmental acclimatization. To aid relaxation, soothing natural sounds were played via a wireless headset. Physical examinations were performed to collect BP, HR, and respiratory rate data. Heart and respiratory rates were measured by employing auscultation with a sphygmomanometer, while BP was recorded using an MTP meter (Medisana International, Neuss, Germany). Body temperature was assessed under the armpits of the left arm using a digital thermometer TM700 (Medisana International, Neuss, Germany). Recommendations were provided to abstain from smoking, intake of stimulants like coffee, and stressful activities for a minimum of 1 h before measurements. For alcoholic beverages, the abstention period was extended to 12 h before the tests.

Before initiating each measurement, offset values for temperature and CF were documented for at least one minute to accommodate mechanically induced residual force. These offset values were subsequently subtracted from the ensuing CF readings. The experimental measurement module was placed on the fingertips of both hands of each participant at 30-min intervals, leading to two separate measurement sets. The wavelength of 940 nm was used as a reference signal for decision-making.

The protocol followed to record the data was the following:

1. For the initial recording of temperature and CF, the fingertip was positioned on the measurement module without exerting extra force for at least one minute.
2. The CF was progressively adjusted using the adjustment screw until the AC component of the 940 nm wavelength decreased to a negligible amplitude.
3. Once Step 2 was accomplished, the CF was gradually reduced until the pressure applied to the fingertip was released.

### 3.3. Data Analysis

Initial data exploration uses the “HeartPy” toolkit, a Python-based HR analysis tool [30,31]. This analysis calculates the DC and AC amplitudes and the AC/DC ratio for each pulse cycle. The “detect_peaks” module from “detecta” [32] is employed to identify the end-diastolic minima and systolic maxima in each PPG cycle. These points are crucial for extracting the signal envelope, which was smoothed using the Savitzky–Golay filter. The DC component is identified as the low-frequency component (below 0.5 Hz) of the PPG and CF signals. The force sensor is the primary tool for determining CF and acquiring the tonometric signal. This signal is derived by applying a known force to the skin surface, which flattens the superficial arteries against the bone and enables recording the BP waveform at that location. This method is commonly called applanation tonometry [33,34].

For statistical evaluation, Pearson correlation coefficients are calculated to assess the linear association between the DC components of the five MW-PPG signal wavelengths and the DC component of the CF. A complementary statistical analysis uses box chart plots to represent the dispersion and central tendencies of the data. The median is displayed in each box chart. Unless otherwise specified, results are presented as mean ± standard deviation (SD).

## 4. Results

The study begins with a comprehensive analysis of the raw MW-PPG signals, focusing on CF-induced properties and quality variations. It includes a comparative analysis of wavelengths to establish a contextual framework for specific response patterns. Subsequently, how CF affects the DC component is explored, emphasizing the correlation between the two. Also, it examines the evolution of the AC pulse contours throughout the ascending and descending phases of the CF.

### 4.1. Initial Analysis and General Behavior of the Signals

**Figure 2:**
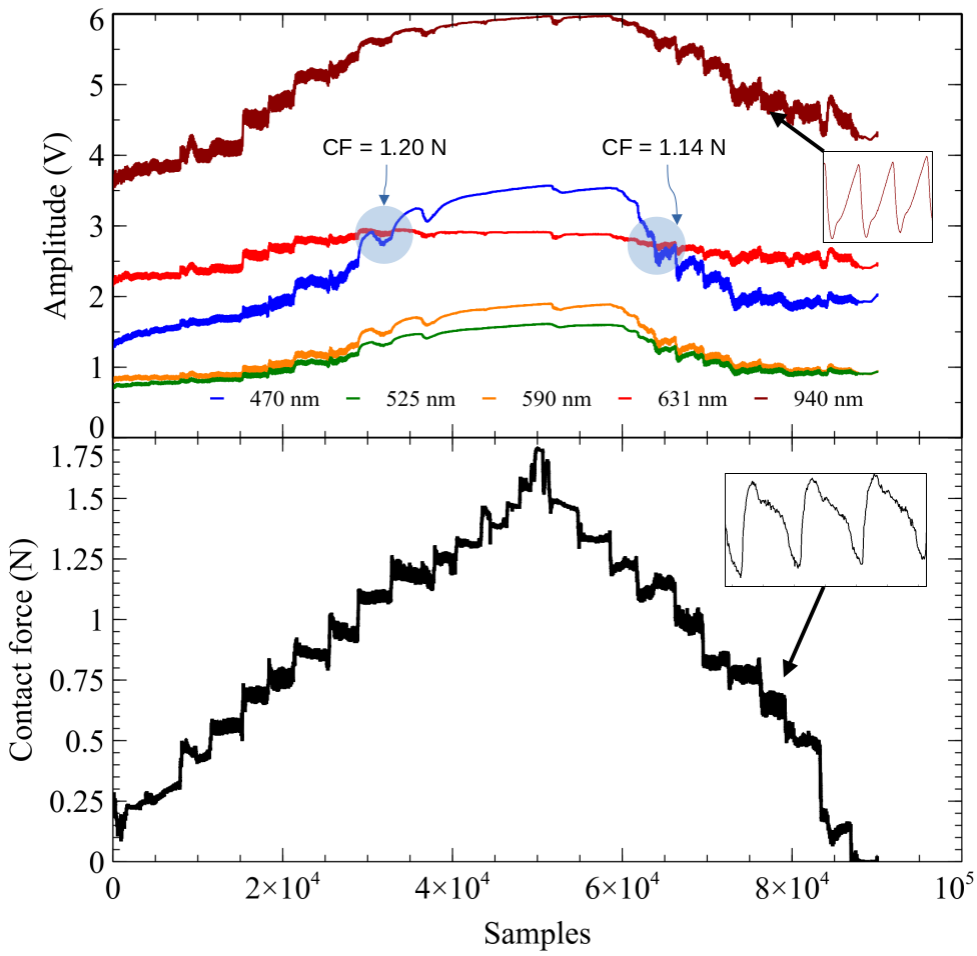
As an illustrative example, the raw signal amplitudes of the SR8 dataset are presented. The five wavelengths of the MW-PPG signal are shown at the top. At the bottom, the increasing and decreasing components of the CF are shown. A sample of the AC component of the PPG signal at 940 nm (**top** image) and the tonometric signal (**bottom** image) are highlighted with small rectangles marked with arrows. The points of convergence between the 470 and 631 nm wavelengths are circled in blue. The CF values corresponding to the convergence points are indicated with blue circles.

An analysis of the raw signals provides an overview of the typical behavior across the dataset. Figure 2 presents the raw signal amplitudes from the SR8 record as an illustrative example. The top panel illustrates the behavior of the five wavelengths, while the bottom panel depicts the CF curve. The tonometric signal captures the effect of the propagation of BP pulses through the skin and underlying tissues. This process results in a detectable local displacement of the skin surface, as illustrated in the bottom panel of Figure 2.

The tonometric signal is present in 17 out of 21 data records. A visual examination focuses on specific criteria to assess the quality of these tonometric signals. Pulses are categorized based on the clarity of the pulsatile component, with signals rated as “excellent quality” displaying distinct and regular pulsations. Another critical factor is consistency in the waveform pattern; signals with high consistency in shape and pattern across multiple cycles are rated higher. Additionally, the assessment considers the amplitude of the pulsatile component and the presence of baseline drift or artifacts. Based on these criteria, six records are categorized as “excellent quality”, five as “good quality”, and the remainder as “poor quality”.

Having established a foundational understanding of the typical behavior across all datasets through analyzing raw signals, as exemplified in Figure 2, the study then explores signal properties and quality variations. In all datasets, the DC component at 940 nm had the highest amplitude, followed by the 470 and 631 nm wavelengths, which had the second-highest amplitudes. Under relatively low CF conditions, in 14 of the 21 datasets, the amplitude of the 631 nm wavelength exceeded that of the 470 nm wavelength.

The apparent convergence of signal amplitudes for 631 nm and 470 nm, indicated by blue circles in Figure 2, is observed at CF values of 1.20 and 1.14 N, respectively. These convergence points were sporadically identified across datasets at low and high CF levels. In particular, convergence during the increasing phase of CF does not consistently correspond to convergence for the same signal amplitude during the decreasing phase of CF.

#### 4.1.1. Wavelength Convergence under CF Variations

**Figure 3:**
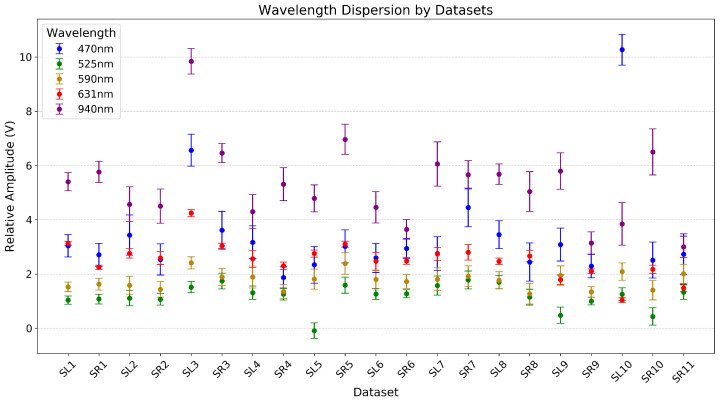
Relative amplitude comparison among data records and wavelengths during CF variation. Data points are displayed as mean ± SD.

To contextualize the subsequently mentioned observations, the scatter plot shown in Figure 3 allows for examining the relative amplitude comparison among data records and wavelengths during CF variation. Upon further analysis of the raw data, we observe additional relative convergence among baseline levels of different wavelengths. In particular, the 631 nm wavelength intersects shorter ones. For example, in the SL9 record, convergence occurs between 631 nm and 590 nm, while in SL10 and SR11, it aligns with 525 nm. For the SR11 record, data from only one right finger could be recorded.

Additionally, convergence is observed between 525 nm and 590 nm in records like SL8, SR8, SR4, and SR7. The behavior of the 631 nm wavelength is unique in that it displays diverse patterns. For instance, in the SL5 record, a triple convergence involving 470 nm, 590 nm, and 631 nm is observed. In SR11, 470 nm intersects with 590 nm during the increasing CF phase and later with 940 nm at higher CF levels. These occurrences are observed during increasing and decreasing CF phases, with 631 nm aligning with 590 nm at lower CF levels. The modulation of the 631 nm wavelength pattern appears less affected by CF variations, evidenced by a less steep slope. This explains why this wavelength converges with others (especially with the 470 nm wavelength), which show a more marked modulation pattern in the face of CF variations.

The convergence observed suggests a differential sensitivity to CF. This may be due to how each wavelength interacts with specific tissue components, such as oxygenated and deoxygenated hemoglobin, and how these interactions change under compression. Compression can alter local blood volume and vascular structure in a way that uniquely affects the absorption and scattering of different wavelengths. The variety in convergence patterns and specific intersection points suggests that the tissue response to compression is not uniform across subjects or even within different regions of the same subject. This reflects differences in tissue composition and vascular density.

#### 4.1.2. Asymmetries in CF across Hands

**Figure 4:**
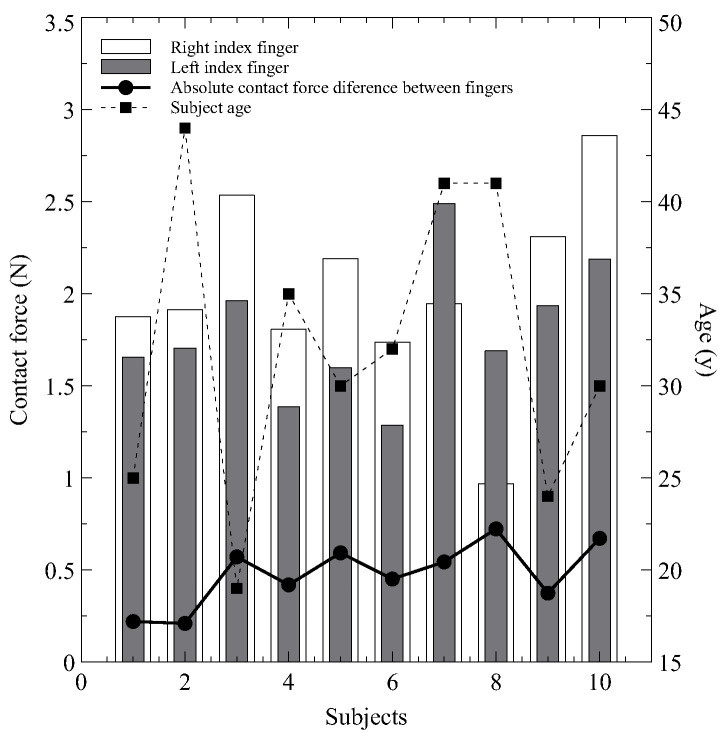
Maximum CF achieved by each participant in the experiment with the reading of the absolute CF difference between fingertips and the age of each subject. Subject 11’s data were excluded from this graph.

Furthermore, Figure 4 presents the maximum CF recorded on the fingertips of each subject, revealing expected biomechanical asymmetries between hands. It indicates that the CF on the right index finger is consistently higher than that on the left in 8 out of 10 subjects, with an average difference of 0.48 ± 0.17 N. This natural asymmetry is potentially due to biomechanical factors such as variations in the anatomical structure and biomechanics of the hands and fingers. However, the dominant hand of the subjects was not recorded during data acquisition. Future research should consider registering the dominant hand of participants.

#### 4.1.3. Local Temperature Variations Due to CF

**Figure 5:**
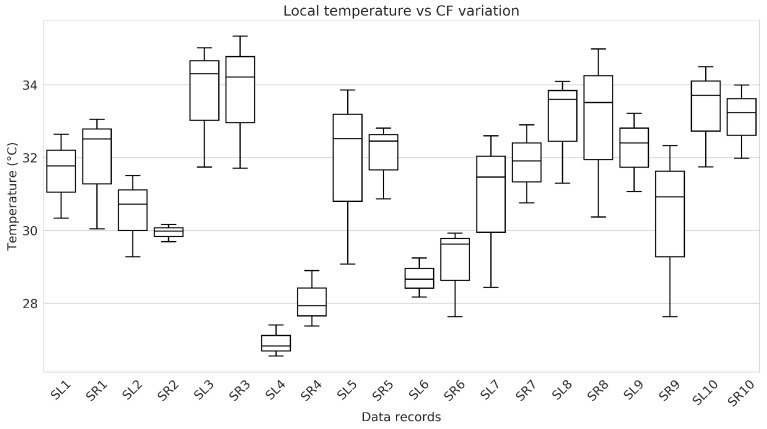
Box plots represent the distribution of local temperature variation per data record, spanning the entire range of CF variation. This range includes from the minimum to the maximum level of CF, followed by a decrease from this maximum point to the minimum level again.

Local temperature was measured at the fingertips using the temperature sensor integrated into the measurement probe. A notable trend was observed in the relationship between CF and local temperature. As the CF increased, there was a marked decrease in local temperature. Conversely, when the CF was reduced, the local temperature gradually rose, slowly returning to its initial value. This dynamic suggests a direct correlation between the intensity of physical contact and the resultant temperature changes, indicating the physiological response of the body to varying degrees of applied force. The statistical analysis of temperature differences, supported by box plot diagrams in Figure 5, reveals distinct patterns among subjects and between fingers of the same subject. It stresses the diversity in the physiological responses reflected in the local temperature in response to the applied CF.

### 4.2. In-Depth Examination of DC Component Response to CF

**Figure 6:**
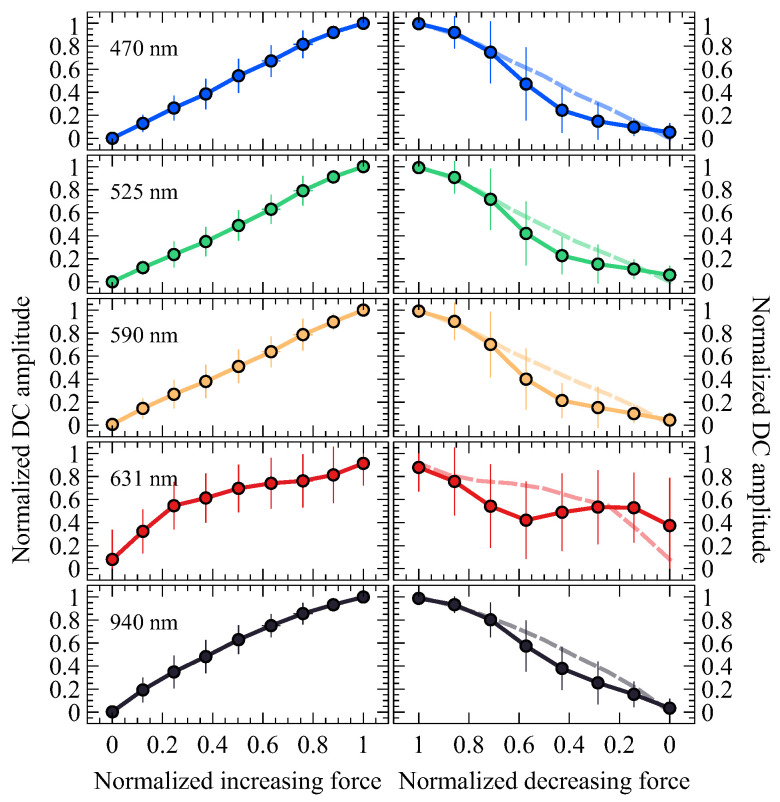
The amplitude of the normalized DC components of each wavelength versus the normalized CF was averaged over all subjects and is presented as the mean ± SD. On the left, the DC components correspond to an increasing CF; on the right, they correspond to a decreasing CF. The dashed connecting line on the right side illustrates the increasing CF transition, with the phase inverted and superimposed for each wavelength for easy comparison.

Building upon the initial analysis that provided a foundational understanding of the general behavior of the signals, the study focuses on a more in-depth examination of how CF affects the DC component of the MW-PPG signals, as detailed in Figure 6.

#### 4.2.1. Analysis of DC Amplitude Trends

Based on the observations from the previous Section 4.1 regarding the unique behavior of the 631 nm wavelength, Figure 6 provides a more precise visualization of this phenomenon, as it showcases the wavelength-specific differences in the DC component response to varying phases of CF. During the increasing phase of CF, a general upward trend in DC amplitude is observed across all wavelengths. However, the 631 nm wavelength exhibits a distinctly different pattern of ascent marked by notable slope changes, setting it apart from the other wavelengths, which follow a more uniform upward trajectory. This divergence is particularly pronounced compared to shorter wavelengths, which have a more linear mean increase. For the 940 nm wavelength, this growth is slightly less linear.

#### 4.2.2. Hysteresis in Wavelength Response

The right column of Figure 6 shows the values during the decreasing CF phase. In addition, the rising phase has been inversed and superimposed to facilitate comparison. This figure highlights the varying paths of different wavelengths through the rising and falling CF stages, revealing hysteresis in these patterns. The distinct behavior of the 631 nm wavelength is particularly emphasized in this visualization. Its amplitude initially decreases, reaching an inflection point at 0.6 of normalized CF, then increases to a second inflection point at 0.2 of normalized CF before decreasing again. This pattern of change contrasts with the more subtle slope variations observed for other wavelengths, further underscoring the unique response of the 631 nm wavelength to changes in CF. In general, hysteresis is an indicator of the ability of tissue and blood circulation to recover from compression. It varies significantly between individuals depending on tissue elasticity, vascular health, and the rate of blood reperfusion. Therefore, hysteresis analysis and comparison of waveforms at the beginning and end of CF decay could provide valuable information on tissue mechanical properties and vascular dynamics. In [28], similar observations are interpreted as evidence of the physiological self-protection mechanism, which increases blood flow in the cutaneous arteries in the face of the risk of occlusion.

#### 4.2.3. Correlation Analysis

Based on the above observations, special attention is paid to the relationship between the DC components of the MW-PPG signals and the CF across the entire range of CF variation. This analysis is predicated on the hypothesis that the modulation trend induced across wavelengths due to variations in CF resembles the CF waveform itself, albeit potentially influenced by non-linear dynamics. To quantify this relationship in each available record, the correlation coefficient is calculated between the DC component of each wavelength and the CF. Hysteresis across all wavelengths suggests variable responses to the CF increasing and decreasing phases. This observation suggests the potential for non-linear correlations that standard correlation coefficients may not adequately capture. The p-value is subsequently employed to determine the statistical significance of the observed correlations, with a threshold for significance set at *p* < 0.05.

**Figure 7:**
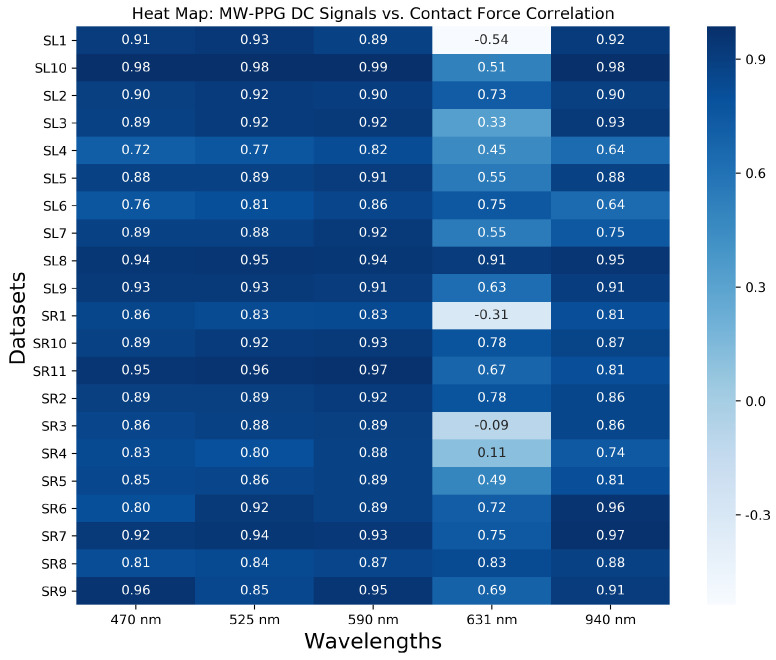
Heat map of Pearson correlation coefficients between DC components of the MW-PPG signal versus the CF.

As illustrated in Figure 7, our findings reveal a generally strong positive correlation across most DC components with CF. An exception is noted again for the 631 nm wavelength. However, the identified hysteresis in all wavelengths calls for more nuanced analysis. This hysteresis implies that the responses to increasing and decreasing CF are not identical, suggesting that our understanding of the correlation must account for these variable responses. While the correlation analysis underscores similarities in trends between the DC components of the wavelengths and CF, it also suggests further investigation into the complex interplay introduced by hysteresis, particularly for wavelengths exhibiting subtler slope changes than the 631 nm wavelength.

### 4.3. Analyzing CF Impact on the MW-PPG AC Component

**Figure 8:**
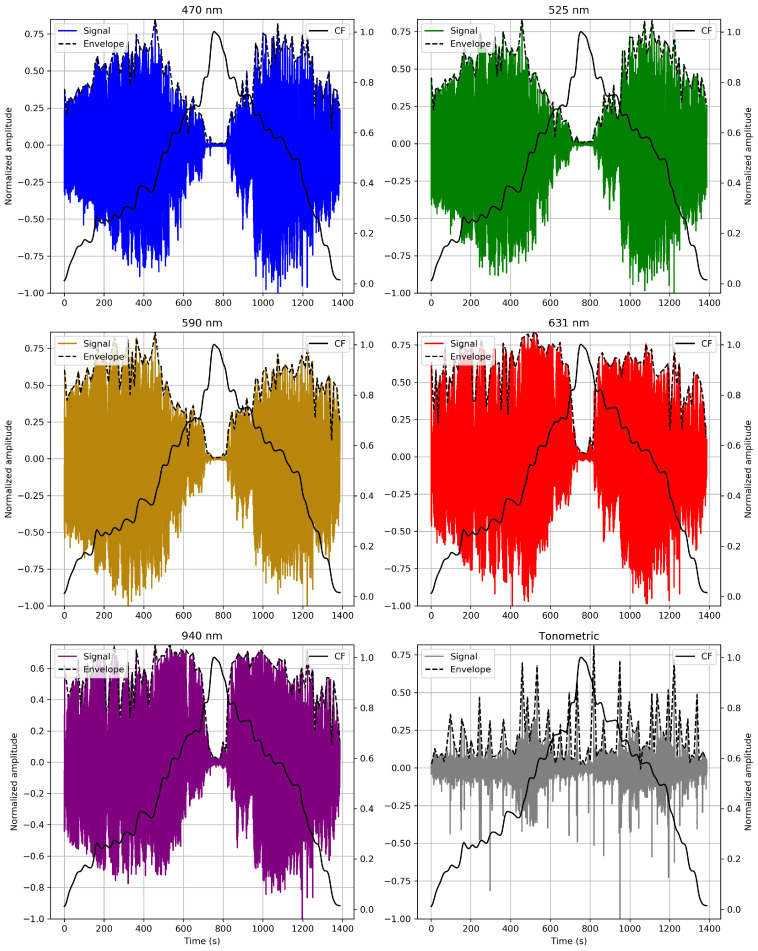
As an illustrative example, the normalized amplitude of the AC component and tonometric signal behavior during CF variations from the SL9 dataset are presented. For comparison, the overlaid normalized CF curve is shown.

To better understand the intricate dynamics of MW-PPG signals against CF variations. The AC components of each wavelength are analyzed. This involves inverting and filtering the PPG signals using a fourth-order analog Butterworth bandpass filter with a 0.5 to 10 Hz cutoff frequency. Figure 8 presents an example from the SL9 record, showcasing the normalized amplitude of the AC component and the tonometric signal during variations in CF. The overlaid normalized CF curve is also displayed for comparative analysis, illustrating the correlation between CF variations and signal amplitude.

During the crescent phase of CF, shorter wavelengths generally reach their maximum amplitude at lower CF levels than longer wavelengths. This could be interpreted as greater sensitivity of short wavelengths to initial changes in CF, indicating an immediate or more direct response to changes in CF. The maximum amplitude of the tonometric signal tends to coincide with the maximum amplitude of the pulses of the 940 nm wavelength. In contrast, during the decreasing phase of CF, different levels of CF are required to reach the maximum amplitude of the AC components per wavelength compared to the increasing phase. This indicates hysteresis in the system and is in line with the observations described in Section 4.2.2, where the response of the wavelengths to decreasing CF is not simply the inverse of its response to increasing CF. This difference suggests that the skin maintains residual deformation from its previous exposure to higher levels of CF, which affects its response to subsequent changes in CF.

#### Evolution of AC Pulse Contours during CF Phases

**Figure 9:**
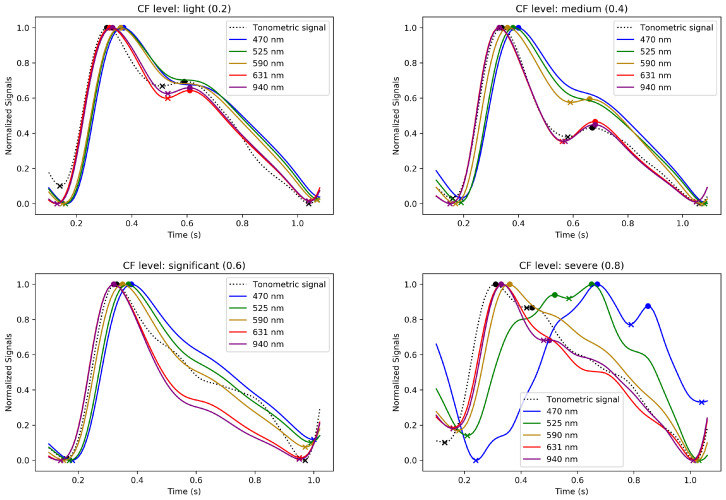
Evolution of the normalized averaged MW-PPG and tonometric signal waveforms during the increasing phase of CF, extracted from dataset SL3. Segments 1 to 4 correspond to normalized CF levels due to light (0.2), medium (0.4), significant (0.6), and severe (0.8) CF levels, respectively. The ‘o’ and ‘x’ markers indicate peaks and valleys of the signal, respectively.

**Figure 10:**
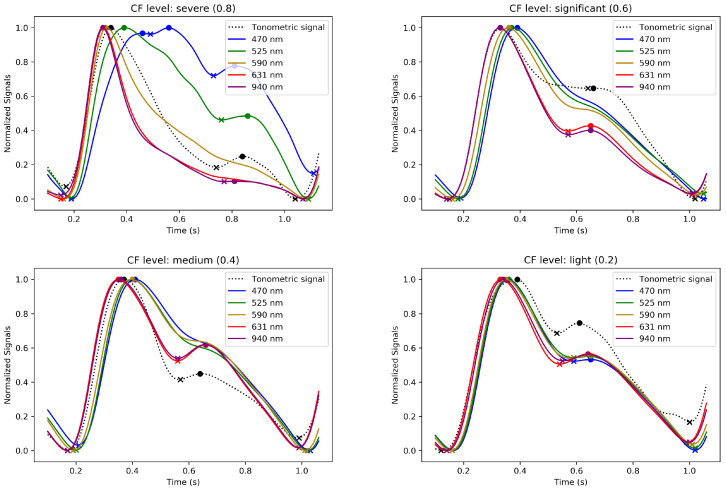
Evolution of the normalized averaged MW-PPG and tonometric signal waveforms during the decreasing phase of CF, extracted from dataset SL3. Segments 1 to 4 correspond to normalized CF levels due to severe (0.8), significant (0.6), medium (0.4), and light (0.2) CF levels, respectively. The ‘o’ and ‘x’ markers indicate peaks and valleys of the signal, respectively.

Figure 9 and Figure 10 illustrate the evolution of the normalized waveforms of the MW-PPG and tonometric signal. These waveforms are observed during the increasing and decreasing phases of CF and are taken from the SL3 record. Additionally, they are displayed across four CF levels. These levels represent a progression of normalized CF ranging from light to severe for Figure 9 and severe to light for Figure 10.

These figures showcase the effect of varying CF on the signal waveforms and illustrate the evolution of the pulse contours in these phases. MW-PPG AC pulses can be classified into two groups according to the depth of light penetration into the tissue: the deep penetration group (DPG) with long wavelengths (631 and 940 nm) and the superficial penetration group (SPG) with short wavelengths (470, 525, and 590 nm). This classification simplifies the analysis of morphological responses of signals.

In the initial stages of the increasing CF phase, especially at light to medium CF levels, MW-PPG signals from the SL3 record exhibit distinct patterns based on light penetration depth. The DPG wavelengths show different behaviors than those of SPG, with tonometric signals aligning more closely with the DPG shape. This alignment with the tonometric signal diverges at higher CF levels, indicating complex vascular responses. Also, the dicrotic notch is more visible in the DPG wavelengths and tonometric signals. As CF increases, the separation in systolic peaks between DPG and SPG wavelengths becomes more pronounced, which may affect measured PPG-based PTT values through the vascular system [25]. SPG wavelengths respond sensitively to compression, morphing into inverted ‘U’ shapes with oscillations in the diastolic phase. DPG wavelengths, on the other hand, form more triangular waveforms with a pronounced dicrotic notch, suggesting a unique aspect of vascular response to CF. Interestingly, beyond this CF level, when the evidence of oscillations or pulsations for SPG wavelengths disappeared, it was still possible to detect pulsatility for the longer wavelengths, especially for the 940 nm signal. However, these were characterized by peaks of very low amplitude, discernible but fluctuating amplitude, and sporadic appearance and were apparently modulated by respiratory movements. This phenomenon was not consistent in all subjects.

On the other hand, during the decreasing phase of CF, a rapid initial recovery is noted, particularly in the DPG wavelengths, followed by a gradual adaptation. Oscillations observed at extreme CF levels also dissipate quickly. The longer wavelengths in the DPG show the dicrotic notch becoming discernible again at significant CF levels. Clustering between the contour variations of DPG and SPG wavelengths remains evident except for at light CF levels, contrasting the response observed during the increasing phase. This hysteresis pattern, as underlined in Section 4.2, reflects the viscoelastic nature of the tissue and is evident in the different responses between increasing and decreasing CF phases. The gradual recovery during the decreasing phase suggests that the tissue does not immediately return to its original state post pressure reduction. This behavior further emphasizes the importance of accounting for the mechanical properties of tissue in the analysis of PPG signals.

**Figure 11:**
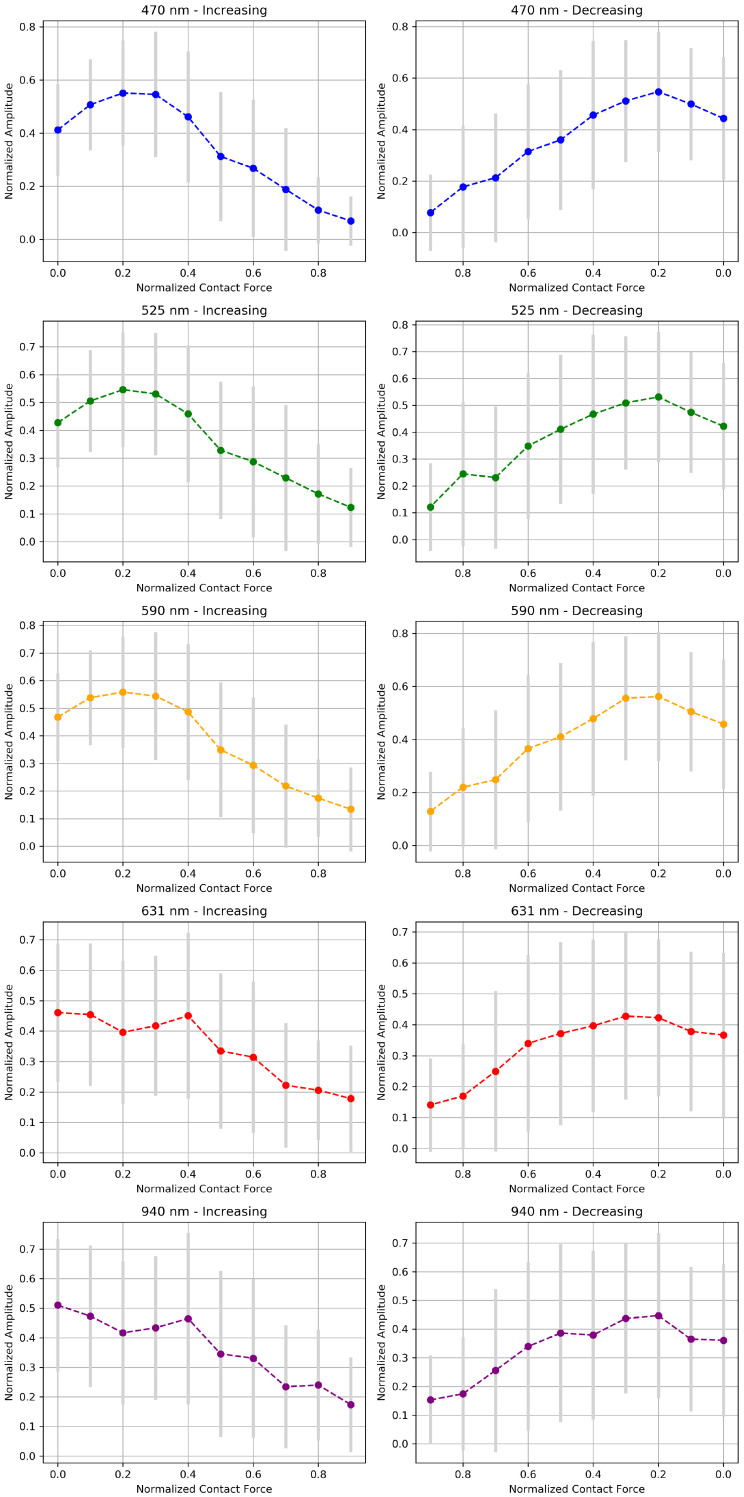
Analysis of the normalized AC amplitude response versus the normalized CF for the five wavelengths for all subjects. Each pair of graphs illustrates the mean ± SD amplitude trends for increasing (**left**) and decreasing (**right**) forces per wavelength.

Figure 11 represents how AC amplitude modulation of MW-PPG signals responds to variations in CF using normalized data to enable consistent comparisons between subjects. The graphs on the left highlight the interaction between normalized amplitude and an increase in CF, contrasting with the graphs on the right that explore the dynamics of decreasing CF. This scheme allows us to appreciate the differentiated response of the SPG and DPG signal groups to adjustments to the CF and also facilitates the identification of hysteresis patterns when comparing the amplitude trends between an increase and decrease in the CF. The analysis highlights the notable inter-individual variability marked by the SD and distinguishes segregation in sensitivity to CF between the SPG and DPG groups.

## 5. Discussion

Our study confirms that CF significantly affects the morphology of the PPG signal depending on the wavelength, individual differences in physiological responses, and skin properties. The selection of wavelengths used in this work takes advantage of the light absorption properties of different tissue components, allowing differentiated evaluation of skin microcirculation at various depth levels. The wavelengths of 470, 525, and 590 nm are applied to study capillaries, which are superficial and require wavelengths that do not penetrate too deeply but are sufficiently absorbed by the blood in small vessels. To observe arterioles and venules, which are located at an intermediate depth in the skin, the 631 nm wavelength is used. Finally, the 940 nm wavelength can reach and be reflected off deeper vascular structures, such as subcutaneous veins and arteries. The index finger was chosen due to its accessibility and consistency as a measurement point for placing PPG sensors in clinical contexts. To increase the reliability of the measurements, they were carried out on subjects at rest and in a controlled environment free of external influences such as movement, electromagnetic interference, etc.

The results have shown that the interaction between the CF and the underlying blood vessels through the skin triggers a series of physiological responses for which the complexity has been emphasized by recent advances in this field [21,35,36]. Previous studies mainly focus on interactions affecting the AC component of the PPG signal, which is crucial for analyzing blood pulsations. Exploration of the DC component opens new avenues for potential applications by considering the contribution of all non-pulsatile components of the finger tissue, including the dermal vasculature from the deep vascular bed to the papillary dermis [37,38]. Moderate levels of CF appear to benefit the AC component, especially at longer wavelengths, where contour sharpness increases, consistent with previous research indicating improved optical coupling between the device and the skin [39]. Yet applying a high CF obstructs blood flow and collapses the capillaries, resulting in pronounced alterations to both components of the PPG signal [39]. This extreme condition reflects reduced detected blood volume and changes to blood flow dynamics, particularly in superficial blood vessels. In complete occlusion of the upper dermis, the pulsation of the deep plexus and subcutis stands out with a significantly stronger contribution. Likewise, DC amplitudes progressively increase, reaching an asymptotic value at higher forces as the blood volume, the diameter of blood vessels, and the hemoglobin concentration at the measurement site are minimized. Consequently, absorbance is reduced while the amount of light detected by the photodetector is maximized. The decrease in CF leads to restoring normal conditions, with the DC component recovering its basal levels and the AC component improving in amplitude and clarity [37].

Hysteresis, observed as a difference in the signal path during the increase and decrease in CF, can be attributed to the non-linear response of capillaries and the dermal blood network to being compressed. The sequence in which blood is expelled from capillaries and blood vessels at different depths results in changes in light absorption that are not immediately reversible when the pressure is released, reflecting the viscoelastic nature of skin tissue and its rheological properties. The signal at 631 nm presents different behavior compared to other wavelengths: presenting significant variability between subjects with pronounced slope changes and even inversion of this slope for specific CF ranges. This peculiarity suggests a unique interaction of this wavelength with tissues through their optical properties and vascular dynamics, which has proven difficult to explain comprehensively. However, it could indicate a differentiated behavior in the face of changes to the oxy–hemoglobin concentration induced by compression. Also, compression could affect the regions this wavelength can explore, altering how DC signals are captured. This reflects a complex interaction that combines the optical properties of the tissue, vascular dynamics, and compression mechanics. These results suggest that light–tissue interactions and vascular dynamics are neither linear nor predictable simply based on penetration depth. This finding underscores the importance of understanding how light interacts with biological tissues in more detail. Variations in tissue composition, vascularization, and oxygenation between individuals or anatomical regions can significantly affect the modulation of PPG signals at different wavelengths. The presence of overlapping signals from multiple depths suggests that the interpretation of PPG data could be improved by more complex models that consider theoretical light penetration, tissue optical properties, and blood flow dynamics.

The correlation between the tonometric signal and the AC component of deep-wavelength PPG signals, especially at 940 nm, during the CF increase phase accentuates the importance of mechanically induced mechanisms in modulating the PPG signal. This alignment indicates that arterial pulse pressure oscillations, which influence capillary blood volume and tissue deformation, determine the formation of the surface PPG signal. This perspective indicates a fundamental interaction between vascular mechanics and tissue optical responses [40,41]. This concept gains further support from research exploring how BP variations and pressure waves influence both blood volume and tissue optical properties, underlining the importance of BP in PPG signal generation [42,43,44].

The potential of using the DC component as a CF indicator to simplify instrumentation, despite needing rigorous validation and consideration of external factors affecting signal integrity, represents a promising direction for reducing measurement costs [23,45]. The results show that shorter wavelengths could be more suitable for this task. Additionally, the CF-induced compression effect on local temperature, attributed to restricted blood flow and influenced by thermokinetic principles [46], reveals the complex physiological impacts of CF.

This analysis emphasizes the need for a comprehensive approach to interpreting PPG signals that considers individual variability and light–tissue interactions. It reveals how a multidisciplinary approach encompassing optics, physiology, and tissue biology is essential to advance the use of PPG in diagnosis and monitoring and points towards more personalized and precise interpretations. It underlines the importance of more refined models that compensate for the complex dynamics of blood circulation and the mechanical properties of tissues. Furthermore, it emphasizes how signal modulation by mechanical variations and BP can deepen our understanding of PPG signal generation, which is crucial for applications in vascular medicine. This approach could improve diagnostic accuracy and open new research directions that focus on developing methodologies that adjust or mitigate these complex effects for better assessment of vascular health and hemodynamic monitoring.

A primary study limitation is the manual CF regulation, which impacts the uniformity of CF changes. This manual approach led to asymmetry in the CF increasing and decreasing phases, yet it did not detract from the study’s findings.

## 6. Future Research Directions

The findings underscore the need for a comprehensive analysis of force-related factors, including the consistency and distribution of the contact force and applied pressure, for evaluating MW-PPG signals. Future research will broaden the scope and number of participants to include a variety of physiological conditions, ages, and health statuses, enabling a more detailed exploration of the impact of CF on signal waveforms and its association with feature evolution. Gaining a deep understanding of how pulse shape alterations influence signal accuracy and reliability, particularly regarding signal quality indices (SQIs), is critical for enhancing the efficiency and precision of PPG technology in healthcare.

Additionally, future research will focus on how CF affects other parameters, such as the AC/DC ratio of the signal, and its influence on the accuracy of measurements of specific physiological parameters, such as the measurement of SpO_2_. This approach will enable a complete understanding of MW-PPG signal dynamics and its application in accurately monitoring vital indicators, thus contributing to significant advances in non-invasive and personalized health monitoring.

## 7. Conclusions

In our study into the impact of contact force (CF) on multi-wavelength photoplethysmography (MW-PPG) signals, data from 11 volunteers revealed a marked, wavelength-dependent influence of CF on signal characteristics. The 631 nm wavelength exhibited distinctive behavior under varying CF levels, indicating the need for deeper exploration into the impact of tissue and vascular structures on PPG signals. Additionally, a detected hysteresis effect suggested a memory phenomenon within the skin, which was influenced by CF and affects signal integrity. Our findings align with existing theories on forming PPG signals influenced by arterial pulse pressure oscillations and light penetration depth. Exploring the DC component as a potential CF indicator emerged as a promising avenue for simplifying PPG instrumentation despite challenges in ensuring accuracy and accounting for external factors. Despite some variability introduced by manual CF regulation, our study marks a step forward in understanding MW-PPG signal dynamics. It sets the stage for future research to enhance cardiovascular monitoring accuracy and reliability in clinical settings.

## Figures and Tables

**Table 1:** Subject baseline data (data are presented as mean ± SD).

Parameters	Values
Age (y)	32.1 ± 8.2
Gender (M/F)	8/3
Heart rate (bpm)	69.55 ± 7.20
Systolic BP (mmHg)	117 ± 12.33
Diastolic BP (mmHg)	70.64 ± 11.43
Body temperature (°C)	36.1 ± 0.48
Skin tone (Subject No.)	II (1), III (2, 3, 7, 8, 11), IV (4, 9), V (5, 6), VI (10)

## Data Availability

Data are contained within the article.
